# Supplementary material for: What part of the brain is involved in graphic design thinking in landscape architecture?
Source: PLoS One. 2021 Dec 23;16(12):e0258413. doi: 10.1371/journal.pone.0258413 (PMC8699602; doi:10.1371/journal.pone.0258413)
Supplement: Attachment — Submitted filename: Response to Reviewers.docx [file pone.0258413.s001.docx]

**Response to Reviewers**

**Editor’s Comments:**

Please pay particular attention to addressing Reviewer 1's concerns regarding the definition of key terms and explanation of the methodology. Providing these clarifications will be essential for a more thorough examination of the results and their interpretation.

**Response:** We would like to express our sincere gratitude to you and to the reviewers. The introduction is reorganized to make the research question clearer. We narrowed and focused the research question to graphic thinking in the landscape design process in the ‘Introduction’ section, and provide more detailed information about our task in the experiments to explain the methodology in ‘Materials and methods’ section.

1. Please ensure that your manuscript meets PLOS ONE's style requirements, including those for file naming.

**Response:** We would like to thank you for your kindly reminder. We check our manuscript and file naming to meet PLOS ONE’s style requirement.

2. We note that Figure 3 in your submission contains copyrighted images. We require you to either (1) present written permission from the copyright holder to publish this figure specifically under the CC BY 4.0 license, or (2) remove the figures from your submission.

**Response:** Thank you for your kindly reminder. We remove the figures which contains copyrighted images, and use text to describe instead.

3. Thank you for including your ethics statement: "Each participant gave their written informed consent as approved by the Research Ethics Committee, National Taiwan University [approval number 201411HM024]."

a) Please amend your current ethics statement to confirm that your named institutional review board or ethics committee specifically approved this study.

b) Once you have amended this/these statement(s) in the Methods section of the manuscript, please add the same text to the “Ethics Statement” field of the submission form (via “Edit Submission”).

**Response:** The ethics committee in manuscript and ‘Ethics Statement’ field of the submission form are amend.

**Reviewer #1**:

The manuscript describes an fMRI study of 24 designers engaging in various aspects of the landscape design task. It is concluded that "designs involving more graphic thinking exhibit significantly more activity in left prefrontal cortex."

This is a potentially interesting manuscript but is not currently ready for publication.

Issues that need to be addressed:

1. The introduction is overly broad and uninformative. The authors start by asking "what is creativity?" But do not provide an answer. They cannot provide an answer because there are no good answers to the question. So why ask the question?

**Response:** Thank you for providing this suggestion. We reorganized the introduction to make the research question clearer. The research question was narrowed and focused to graphic thinking in the landscape design process. We first point out the importance of graphic thinking, which serves as the stimulus for creative ideas in lines 31–36 of the revised manuscript, then illustrate the connection between the landscape design process and the brain based on the Frontal Lobe Lateralization Hypothesis in lines 37–55. In the end, we state our research purpose of exploring the neural mechanisms associated with the graphic thinking process by using the fMRI scan to identify which part of the brain predominates during this phase of landscape design in lines 59–73.

2. They introduce the notions of without explaining them. I do not know what these terms refer to. They then refer to some findings regarding creativity, but it is not clear what type of creativity they are referring to.

**Response:** Thank you for this comment. After we narrowed the scope of this research, we used terms related to creativity with caution. This research focused on graphic thinking instead of creativity to describe the notion of the landscape design process in the restructured introduction.

3. More generally, the manuscript is about "graphic thinking"… So why start talking about creativity? The discussion is essentially vacuous and unnecessary.

**Response:** Thank you for the suggestion. In the revised manuscript, we deleted unrelated discussion about creativity.

4. Their references regarding problem-solving are dated.

**Response:** Thank you for the comment. As unrelated discussion about creativity was deleted, we removed indirect references, including in the problem-solving section.

5. I do not understand what they did with the Torrance test.

**Response:** Thank you for pointing this out. In the revised manuscript we have included further explanation to address these comments. The Torrance Test is our evaluation tool for verifying the results of brain activation with behavioral performance. Although we use fMRI to investigate what part of the brain was involved in the design process, it is important and needed to validate these brain activity results with other indicators. The methods for evaluating the creativity level of graphic thinking are provided in the section ‘2.5 Evaluation of graphic thinking scores’ (lines 141–166).

6. There are some methodological shortcomings that do not have an easy solution. In particular, what is graphic thinking? What cognitive activities are actually being measured? On the one hand the authors are talking about problem solving in terms of problems scoping, preliminary solutions, refinement, and detailing (figure 1). On the other hand, their task involves viewing, copying drawing, brainstorming, graphic thinking (figure 4). How does the one vocabulary map onto the other? Even more importantly, what is happening during the 60 seconds allowed for each phase? What are the participants doing? What cognitive processes are being engaged? What behavioral data can the authors provide to support their conjecture? There is no way of knowing based on the information provided. If we cannot answer these questions, we cannot even begin to interpret the results.

**Response:** Thank you for the suggestions. We have reorganized our manuscript and hope our revised and edited version is clearer for readers. In this manuscript we define graphic thinking based on Laseau (2000) as thinking assisted by sketching, a process that helps to stimulate ideas (lines 32–34). We describe the design process (Fig. 1) in more detail in lines 37–55 to illustrate the stages in which graphic thinking occurs, and we define the task of brainstorming and graphic thinking we designed in ‘2.4 Procedure’ (lines 113–116). The brainstorming period serves as the preliminary solution step, and graphic thinking is represented in the refinement stage. In addition, the control task of viewing and copying drawings were designed to represent a lack of design process (Fig. 4, lines 116–125).

further description were added to clarify what the participants were doing during each phase in the experiments in lines 126–131. We designed and analyzed the tasks to answer the research question considering the following aspects:

(1)	In this study, we got participants’ real drawings and designs on the paper to serve as evidence of their behavior performance and to make sure participants did the design in the experiment. Moreover, a sanity check was conducted to confirm that the participants’ brain activity results were credible. The primary motor cortex in the brain was activated when participants engaged in hand movement (‘3.1 Sanity check’) (lines 209–213).

(2)	The participants were asked to actually draw their design on the paper in fMRI. Since we had the participants’ design diagrams, we had behavioral performance for verifying our brain activation results.

(3)	The main purpose of this study was to find out what part of the brain is involved in graphic thinking. Since graphic thinking helps to produce ideas and develop creative design, we tried to show the relationship between the graphic thinking area of the brain and the graphic thinking score (using creative thinking indicators). The result showed a positive association between them (lines 281–286, 307–310). Creativity could be involved in this process.

**Reviewer #2:**

This paper addressed a very important question about the brain's underpinning of human behavior, specifically creativity and graphical design. The methodology and results are convincing, and the discussion is to the point. As far as this Reviewer is concerned this version can be published as is.

**Response:** Thank you for your time and energy in reviewing our manuscript. We appreciate your comments and affirmation.
