# Supplementary material for: What part of the brain is involved in graphic design thinking in landscape architecture?
Source: PLoS One. 2021 Dec 23;16(12):e0258413. doi: 10.1371/journal.pone.0258413 (PMC8699602; doi:10.1371/journal.pone.0258413)
Supplement: Attachment — Submitted filename: Response to Reviewers.docx [file pone.0258413.s002.docx]

**Editor’s Comments:**

The manuscript has been re-evaluated by two reviewers, and their comments are available below. You will see Reviewer 2 has commented in the improvement of the manuscript. However, Reviewer 3 has raised critical concerns and the manuscript will need significant revision before it can be considered for publication – you should anticipate that the reviewers will be re-invited to assess the revised manuscript, so please ensure that your revision is thorough. I have outlined some of the key concerns noted by the reviewers below, but you should respond to all concerns mentioned by the reviewers in your response-to-reviewers document.

The key concern noted by Reviewer 3 relate to the definition of terms, the need for additional supporting references, and clarification regarding the fMRI analysis of the ventricles. These issues impact the interpretation of the results and should be explored.

**RESPONSE:** Thank you for the opportunity to revise our paper once more for PLOS ONE. We have modified several points, such as using “Graphic design thinking” thoroughly, added supporting references to explain the relationship among graphic design thinking, landscape design, and the brain, and clarified the results in fMRI analysis. The revised version is shown in gray blocks. We hope the revised version provides more details and gives a clearer structure for readers.

**Reviewer 2 Comments:** The authors have addressed the use of the term creativity, which made the revised paper more scholarly than the original version.

**RESPONSE:** Thank you for spending your time and energy reviewing our manuscript. We appreciate it.

**Reviewer 3 Comments:** This paper analyses the brain areas activated in a design task where designers were asked to perform a landscape design task under the fMRI scanner in two stages: brainstorming without drawing and graphic thinking where they draw and refine the ideas they generated during the brainstorming stage. There were two control conditions were of viewing a plan and imagining being there and another was to copy a plan. I have reservations about the design of the study and the depth of conceptual underpinnings of the study. Most of the terms are loosely defined and leads to confusion. The introduction and discussion are not detailed and severely lack references.

**RESPONSE:** We would like to express our sincere gratitude to the reviewer. Your insightful comments led us to think deeper throughout the revision of this article. We have revised “graphic thinking” to “graphic design thinking” and clarified the terms “design thinking” and “graphic design thinking” as related to architectural design. Besides, we also added several references to support our research. The following is our point-by-point response to your questions and comments. Revisions may be found in gray highlighted text in the revised manuscript. We appreciate your time and effort. Thank you.

**Reviewer #3**

1. The title of the paper, “What part of the brain is involved in graphic thinking for landscape design?” is misleading. This insinuates a specific part of the brain is involved in graphic thinking, specifically for landscape design. This is not true for many reasons, but the most pertinent is that the authors have not checked any other graphic thinking in any other creative fields, like book cover design or jewelry design or product design. Some comments on the commonality and differences between the other kinds of graphic thinking would have put this study in perspective.

**RESPONSE:** Thank you for providing these insights and suggestions. We agree with you and have incorporated your suggestions. We minor revised our title to “What part of the brain is involved in graphic design thinking in landscape architecture?” In different fields of design, the design process could be similar. However, it would be too broad to compare different types of graphic thinking. The study specifically focuses on landscape design and brain activation; therefore, in the beginning of the first paragraph of the Introduction, we have revised these points with the following statements: Design thinking is a highly cognitive activity that is widely used in design-related fields to solve the problem in the man-made environment. “Design methodology,” as described by Cross [1], is the design process on work and thinking developed by designers through techniques or methods that reflect the knowledge to solve a problem. “Design as a discipline” involves different perceptions in the sciences, humanities, and design thinking, knowing, and practical methods [2, 3] (lines 35-40).

2. The first line of the abstract is problematic – “Graphic thinking is a key skill for landscape designers, but little is known about the links between design thinking and brain activity.” How are graphic thinking and design thinking related? There is a large body of work done on design thinking, but none of them are cited (later in the article), nor are the connections between the theories in design creativity and graphic thinking for landscape design explained.

**RESPONSE:** We have made every attempt to address these comments in the revised manuscript in the abstract and the beginning paragraph (lines 40–56), which is shown below:

Hence, we could infer that design thinking involves a series of reasoning mechanisms to select, identify, and then solve the problem [2, 4, 5]. This design process is called “real-world problem solving” with the “select-and-combine model” [6], which relates to the mechanism of conceptual sketches of cognition [7] that might support the frontal lobe lateralization hypothesis (FLLH) in brain activations [4]. Gero and Milovanovic [5] considered that design thinking involves cognition, and researchers have tried to explore the design process through “protocol analysis,” “Black Box experiment,” and “survey” during design. Goel [4] demonstrated architectural designs that included initial sketching with ambiguous lines, conceptual transformation, analysis, and adjustment of the spatial form, gradually converging the style and precision of its spatial structure to solve current problems. By contrast, Gillieson and Garneau [8] highlighted that graphic design thinking as a design process uses visual communication, such as organization and logic, to define deductive and inductive thinking in space, which also involves personal experiences to recall, reframe, and solve the design problem. Their study addressed the design process by drawing a series of graphics to identify the space in the landscape. Therefore, in this study, we used the term “graphic design thinking” to explore the “Black Box” in landscape architecture design.

Moreover, we have clarified our research purpose in lines 109–117 (see below):

During design, designers use sketching to draw preliminary ideas and refine them. Landscape architecture design deals with landscape elements, such as water, plants, and pavements, to integrate elements into a whole that form the compatibility in the space. Architectural design involves structures and coordination in buildings. The design process might be similar. A landscape architecture design involves problem structuring, preliminary design, refinement, and detail, which, as proposed by Geol [7], could represent the graphic design thinking process of design with different elements in the landscape. Using fMRI could shed light on the “Black Box” in the brain activation of landscape architects during the design process.

3. The introduction lacks scholarly depth and references.

**RESPONSE:** We agree with the comments and thank the reviewer for the constructive criticism that we did not make in-depth descriptions in the introduction. We have revised the introduction with several supportive references about design thinking, graphic design thinking, landscape architecture design, and brain. Moreover, we added a paragraph discussing design thinking in architecture and interior design in the brain (see lines 89–108).

4. It is not clear what the authors describe as “graphic thinking.” The definition, without elaboration; “thinking assisted by sketching” is not adequate. It also implies that sketching and thinking are separate, and that sketching can occur without thinking? Although it might be true in common parlance, sketching “without thinking” also is a complex cognitive task. Also, how does graphic thinking differ from visual imagery, visual creativity etc.

**RESPONSE:** We honestly feel that your insightful suggestions helped us improve the concept in our manuscript. The statement of “thinking assisted by sketching” was revised as follows: Laseau [9] described a thinking process assisted by sketching, while Geol [7] identified four characteristics in design, including problem structuring, preliminary design, refinement, and detail, which use sketching to refine ambiguous ideas in the related arts (e.g., architectural design). This suggests that designers think through sketching to visualize and spatialize a concept map within incomplete ideas and refine those thoughts through abductive thinking [10] (lines 57–62). Based on the concept of sketching-assisted thinking proposed by Laseau [9], Geol [7], and Kolko [10], we might infer that, for landscape architects, the landscape architecture design process is associated with the conceptual stages of a project, as landscape architects use sketching to nurture creative ideas, and graphic design thinking refines the ideas to develop and connect landscape elements for completion (lines 69–73).

Moreover, the statements about graphic design thinking and visual imagery are shown below:

The right PFC is involved in planning, visual processing, or reasoning out of the initial concept and preliminary ideas. The right PFC contributes to the abstract, vague, and conceptual aspects of performance. By contrast, after selecting a specific solution and proceeding to further refinement, the left PFC assists in processing specific, clear, and practical information (Fig 1) [4] (lines 82-86); The DLPFC and ACC are not only involved in executive functions, such as visual imagery and semantic processing, but also work together to construct new ideas that respond to problem solving in design tasks [12, 15] (lines106–108).

5. There are no references provided for the argument that “The landscape design process contains four steps” lines 37-46. This is vital.

**RESPONSE:** Thank you for the suggestion. The original sentences in lines 37–46 were not clear; therefore, we revised them as below; please see lines 62–69.

Landscape architecture design consists of a set of design processes, including the scope of the problem, the purpose, and the goal, which interprets “what” and “how” through the selection of material, landscape elements, colors, etc. in the design process to meet needs and create values [11]. The abductive thinking helps designers to develop ideas and connect elements for thought completion [10]. Moreover, the voices of potential users and related stakeholders are important [11]. After the development of the selected solution, the specific idea for actualizing the detailed design will be considered complete.

6. There are no references for “In real-world problem-solving, the left and right prefrontal cortices (PFC) are responsible for different functions.” Lines 49

**RESPONSE:** Thanks for the suggestions. We added the reference and revised the sentences as follows: Goel [4] proposed the FLLH, in which the left and right prefrontal cortices (PFC) are responsible for different functions in real-world problem-solving. The hypothesis links the design cognitive process to brain activity in architectural design and planning [13] (lines 79–82).

7. “The landscape designer develops a concept through brainstorming and then processes ideas, using graphic thinking to refine the design” Lines 54 – There are too many terms here that are not well defined – brainstorming? What does that mean? How is it different from “processes ideas”?

**RESPONSE:** Thanks for the comments. We agree with your suggestion that too many terms were not well defined. We deleted the term “brainstorming” because we considered it to be the design process of producing or refining ideas. Therefore, the original statement in line 54 was revised into the below statement, lines 69–73:

Based on the concept of sketching-assisted thinking proposed by Laseau [9], Geol [7], and Kolko [10], we might infer that, for landscape architects, the landscape architecture design process is associated with the conceptual stages of a project, as landscape architects use sketching to nurture creative ideas, and graphic design thinking refines the ideas to develop and connect landscape elements for completion.

8. “Although Goel advanced this hypothesis to explain the neural mechanisms underpinning the design process, there was no empirical evidence to validate this assumption.” Lines 61. This is not true, Goel has published lesion-studies (including an architect with a lesion) and brain imaging studies to support his theory.

**RESPONSE:** Thank you for pointing this out. In the revised manuscript, we deleted the original sentence in line 61. We have included supporting researches to address these comments in the new paragraph in lines 74–82 and lines 89–108 as below:

A study proposed a framework of using electroencephalography (EEG), functional magnetic resonance imaging (fMRI), and functional near-infrared spectroscopy (fNIRS) to measure brain activation, which could offer a glimpse of the design process, such as design creativity, design reasoning, and problem-solving, during different design tasks [5]. fMRI may allow a better understanding of design cognition, such as visual and spatial reasoning, in design thinking and creativity [12]. Goel [4] proposed the FLLH, in which the left and right prefrontal cortices (PFC) are responsible for different functions in real-world problem-solving. The hypothesis links the design cognitive process to brain activity in architectural design and planning [13].

The involvement of the PFC in cognitive mechanisms has been proven in neuropsychology [14]. Related research on architecture and interior studies in design results linked with brain activation. The specific design tasks replete with sketching the frame and design items in space in various forms prompted our research design. In their study, Goel and Grafman [13] demonstrated that a patient with frontal lobe lesions who was an architect was unable to perform a preliminary design in sketching the ideas. However, normal subjects could perform the preliminary design, refinement, and detail through the design process in the open-end design. The finding highlights the role of the right PFC in design thinking tasks, consistent with other findings on interior design [12, 15, 16]. Alexiou et al. [12, 15] showed that when subjects in their study used a trackball mouse to move objects during interior design tasks (with no restriction), certain brain areas (e.g., dorsolateral prefrontal cortex (DLPFC), anterior cingulate cortex (ACC), middle frontal gyrus, and middle temporal gyrus) were more active than during problem-solving tasks (with the conditions). These results were consistent with those of Gilbert et al. [16], who found that the right DLPFC and left frontal lobe were more active, stimulating potential solutions within visual imagery in design tasks. Further, these studies indicated that during design tasks, the activation of ACC could relate to cognitive and emotional functions [12, 15]. The DLPFC and ACC are not only involved in executive functions, such as visual imagery and semantic processing, but also work together to construct new ideas that respond to problem solving in design tasks [12, 15].

**Procedure**

9. “brainstorming, using geometric illustrations to envision a landscape design (idea generation) (B-1); and graphic thinking, using drawing to modify the design from the brainstorming session (idea production) (B-2)” lines 122 - We have no idea what the participants were doing in the brainstorming session. So, it is wrong to say that the designers are modifying the design from the brainstorming session. They might be producing the ideas and drawings together, as claimed by the definition of graphic thinking “thinking assisted by sketching”

**RESPONSE:** Thanks for the comments. This is a good point that we could clarify our term in research procedure. The following is the revised statement:

The landscape graphic design process includes preliminary ideas, in which the participants think of initial solutions to the refinement problem-solving step. In the refinement phase, the subjects drew and refined their ideas during graphic design thinking, which we assumed activated the left PFC (lines 160-163); “preliminary ideas,” using geometric illustrations to envision a landscape architecture design (idea generation) (B-1); and “refinement” of the ideas, which uses designing skill to produce the preliminary ideas, reasoning, and drawing of landscape elements (idea production), consistent with the description of graphic design thinking assisted by sketching (B-2) (lines 168-172).

10. “In the “viewing” condition, participants were asked to imagine they were in the environment that the plan presented.” - Lines 126 – This is not a simple viewing condition, but a complex cognitive task of transporting oneself into an imaginary place. This aspect has not been interpreted correctly in the results nor explained in the discussion.

**RESPONSE:** Thanks for the comments. We agree that the “viewing” condition involves not only viewing but also visual imaginary a place. That is also why we used it as a contrast to find out which brain is involved in generating ideas in the preliminary ideas phase. We explain it more clearly in lines 249–253:

First, we compared the difference between “preliminary ideas (B1)” and “viewing (A1)” to understand the design brain of provide preliminary ideas. Both of these two conditions involve the cognitive brain, which includes visual imagery to transport oneself into the landscape, but the “preliminary ideas” condition also engages the brain in generating ideas.

11. “To avoid any ordering effect, the two tasks were run as ABAB BABA or BABA ABAB” - lines 137. This kind of design does not remove the ordering effect as A always follows B, only the starting condition has been changed. To truly remove the ordering effect, the presentation must have been randomized.

**RESPONSE:** We apologize for the unclear statement. We tried to use two versions of the task sequence to reduce the ordering effect. We revised the statement as follows:

The participants were randomly assigned to one of the counterbalancing task sequences, ABAB BABA or BABA ABAB (Fig 4) (lines 186-187).

The ordering effect issue was added as a limitation in “Discussion and Conclusions” (lines 350-352): Moreover, limited to the preprint booklet, the task sequence in this study has only two counterbalancing versions. It is better to randomize the experimental tasks to avoid ordering effect.

**Results**

12. “The results show brain activation at the lateral ventricle, which contains cerebrospinal fluid that protects the brain from impact injury and assists in nutrient cycling and waste removal. It is not responsible for specific cognitive function. On that basis, our hypothesis that the preliminary solution (idea generation) phase activates the right PFC was not verified” – 254. I am confused by this result. How can hemodynamic activity be seen in the ventricles? I am not an expert in fMRI analysis, but this is very suspect.

**RESPONSE:** Thank you for pointing out this problem. We carefully recheck and reference from “Farthing, J. P., Cummine, J., Borowsky, R., Chilibeck, P. D., Binsted, G., & Sarty, G. E. (2007). False activation in the brain ventricles related to task-correlated breathing in fMRI speech and motor paradigms. Magnetic Resonance Materials in Physics, Biology and Medicine, 20(3), 157-168.”. The activations in ventricles are (motion) artifacts and are not responsible for specific cognitive function. We would like to remove this ventricle result and revise the discussion of the null result in lines 325–327.

13. “As this brain region is in charge of cognitive processes, including working memory, cognitive flexibility, and planning [10], there is support for the hypothesis that the left PFC is responsible for refining and detailing (idea production)” - This contradicts authors claim in lines 37 to 40. Idea production happens before refining and detailing stage.

**RESPONSE:** Thank you for the comments. There is a little misunderstood. Idea production phase happens in the refinement and detailing stage. The statement is in the revised manuscript lines 120–122: According to Goel [4], the preliminary solution (or idea generation) phase activates the right PFC, whereas the refinement and detailing (or idea production) phase activates the left PFC.

**Discussion**

14. “the only region activated was the lateral ventricle. Further experiments are needed to facilitate interpretation of this finding” 302 – The ventricles cannot show hemodynamic activity.

**RESPONSE:** Thank you for pointing out this problem so that we can make it clearer description in this points. As response 12, we revised the discussion about the null result in lines 323-337. Having controlled for the imaginary landscape by analyzing “preliminary ideas versus viewing” (both of which are needed to imagine environmental space but differ in relation to graphic design thinking), there was no brain region activity shown for this phase in this study. It is possible that the imaginary landscape could not evoke sufficient graphic design thinking that can be captured in the brain result.

15. Besides graphic thinking, this also relates to other elements, such as emotions, memories, and experiences.” Line 315 - please cite references

**RESPONSE:** Thank you for the comments. We added the references, see lines 338-341. The present results confirm that the left middle frontal gyrus is involved in graphic design thinking in landscape architecture design and can be linked to the idea production phase of creativity [4, 12, 13, 15, 16]. Besides graphic design thinking, these findings also relate to other elements, such as emotions and personal experiences [7, 8, 12, 15].
