# Supplementary material for: What part of the brain is involved in graphic design thinking in landscape architecture?
Source: PLoS One. 2021 Dec 23;16(12):e0258413. doi: 10.1371/journal.pone.0258413 (PMC8699602; doi:10.1371/journal.pone.0258413)
Supplement: Attachment — Submitted filename: Response to Reviewers.docx [file pone.0258413.s003.docx]

**Editor’s Comments:**

1. Line 217: use proper capitalization, 3 T or 3 tesla (Tesla => tesla)

**RESPONSE:** Thank you for the suggestion. We corrected the wording to 3T in line 217.

2. Line 218 please double check T1 scan parameters, these does not look parameters suitable for T1 weighting. I’ll check the images on the repository (see below).

**RESPONSE:** Thank you for check the parameters. We checked the parameters in our experiment which was used T2. We corrected this mistake in the manuscript in line 218.

3. Line 221 TE= 30 ms (add "s")

**RESPONSE:** Thank you. We added “s” in the revised version in line 222.

4. Line 222 matrix size=64x64x45, without mm3 unit (these are pure numbers)

**RESPONSE:** Thank you for the correction. We deleted the unit for matrix size in line 222.

5. Line 226 please delete "According to the preprocessing steps in the SPM8 manual" remark. It sounds naive.

**RESPONSE:** Thanks for the suggestion. We deleted the sentence in line 226.

6. I must highlight that the MRI protocol is remarkably old style. It is very odd, especially considering the high performance scanner that was used. Is there any reason behind this methodological choice? You could add a remark like "no acceleration was used to minimize the scans sensitivity to motion", that on my eyes is still unsatisfactory, but at least hints why a 15 yrs old experimental approach was used.

**RESPONSE:** While a shorter TR might reduce sensitivity to motions, the fMRI protocol we adopted was the default protocol recommended by our imaging center to yield a good signal-to-noise ratio with our scanner. For clarification, we have also revised the description of our protocol to include more details in lines 217-224:

“Images were acquired by a 3T SIEMENS MAGNETOM Prisma MRI with a 20-channel head coil. For each participant, a T2-weighted anatomical was obtained (TR = 9530 ms, TE = 103 ms, flip angle = 150°, field of view = 192×192 mm2, x-y voxel size = 0.66×0.5 mm2, 3mm thick). Two-dimensional echo-planar images (EPI) were acquired with a GRAPPA acceleration factor of two at repetition time TR = 3000 ms, echo time TE = 30 ms, flip angle = 90°, field of view = 192×192 mm2, matrix size = 64×64×45, and an effective resolution of 3×3×3 mm3. In total, 45 EPI slices were sampled in a bottom-up, interleaved order.”

7. Sanity check section: please add information on the overall motion of the subjects during the functional scans, eg a table with average framewise displacement in each run.

**RESPONSE:** Thank you for the comment. We added the information on the overall motion of the subjects during the functional scans in sanity check section (lines 261-264 and table 1).

8. Line 350: what is the "preprint booklet"? It is the first time this term appears. Please use the same wording used in methods.

**RESPONSE:** Thank you for the suggestion. We changed the wording of “preprint booklet” to “the experimental tasks printed and bound on paper” in revised version in line 354.

9. Data availability statement is missing in the manuscript, and the version in front matter is misleading ("All relevant data are within the manuscript.", but no raw data is actually available in the manuscript). MRI scans and scores from each subject must be made available on a public repository before acceptance, and the link must be included in the final manuscript version.

**RESPONSE:** We uploaded the MRI scans and scores from each subject to a public repository. We modified the data availability statement as “All relevant data is available on Harvard Dataverse Network (<https://doi.org/10.7910/DVN/ZHZG5C>).”

**Reviewer 2 Comments:** The authors have addressed reviewers' criticisms, comments, and suggestions adequately, and this version of the paper is good now. I recommend acceptance for publication.

**RESPONSE:** Thank you for spending your time and energy reviewing our manuscript. We appreciate it.
